# Positive and negative outcomes of informal caregiving at home and in institutionalised long-term care: a cross-sectional study

**DOI:** 10.1186/s12877-017-0620-3

**Published:** 2017-10-10

**Authors:** Silke F. Metzelthin, Ellen Verbakel, Marja Y. Veenstra, Job van Exel, Antonius W. Ambergen, Gertrudis I. J. M. Kempen

**Affiliations:** 1 Department of Health Services Research, Faculty of Health, Medicine and Life Sciences, Care and Public Health Research Institute (CAPHRI), Maastricht University, P.O. Box 616, 6200 MD Maastricht, Netherlands; 2 Department of Sociology, Radboud University, P.O. Box 9104, 6500 HE Nijmegen, Netherlands; 3 Huis voor de Zorg, P.O. Box 5185, 6130 PD Sittard, Netherlands; 4 Erasmus School of Health Policy & Management, Erasmus University, P.O. Box 1738, 3000 DR Rotterdam, Netherlands; 5 Department of Methodology and Statistics, Faculty of Health, Medicine and Life Sciences, CAPHRI School for Public Health and Primary Care, Maastricht University, P.O. Box 616, 6200 MD Maastricht, Netherlands

**Keywords:** Informal caregiving, Family caregiving, Quality of life, Caregiving burden, Institutionalised long-term care, Community-dwelling older adults, Ageing in place, Home care

## Abstract

**Background:**

Our ageing society is putting tremendous strain on public health and welfare programs to meet the needs of ageing individuals. Promoting informal caregiving is one way for policymakers to reduce this burden. However, caregiving may be experienced as stressful and is associated with adverse health consequences. While quite a lot of research focuses on caregiving for community-dwelling older adults, little is known about informal care in institutionalised long-term care (ILTC). Therefore, the objectives of this study were: 1) to compare characteristics of informal caregivers and care receivers and caregiver outcomes - at home and in ILTC; 2) to study the association between these characteristics and positive and negative caregiver outcomes; 3) to investigate the moderating effect of the setting (at home vs. ILTC) on these associations.

**Methods:**

A cross-sectional study was conducted using the TOPICS-MDS DataSet. A total of 5197 Dutch dyads were included. The average age of the care receivers and caregivers was respectively 80.7 years and 63.2 years. Several sociodemographic, health-related and caregiving-related characteristics of care receiver and caregiver and two caregiver outcomes (i.e., subjective burden and care-related quality of life) were included in the analyses.

**Results:**

Caregivers in both settings experienced comparable levels of subjective burden. Caregivers at home had slightly lower care-related quality of life than caregivers in ILTC. Several care receiver characteristics (i.e., male sex, married/cohabiting, more morbidities/disability, and less self-perceived health/psychological wellbeing) and several caregiver characteristics (i.e., female sex, being younger, living together with the care receiver, more objective burden, less self-perceived health, and more support) were associated with an increase in burden and/or a decrease in care-related quality of life. Some of these associations were stronger for dyads at home compared to dyads in ILTC.

**Conclusions:**

Informal caregiving does not stop with admission to an ILTC facility. Both settings need an informal caregiving policy, which is (1) tailored to the individual characteristics of care receivers and caregivers; (2) pays attention to the identified risk groups; and (3) reduces the negative caregiver outcomes and emphasizes the positive outcomes at the same time.

**Electronic supplementary material:**

The online version of this article (10.1186/s12877-017-0620-3) contains supplementary material, which is available to authorized users.

## Background

Like most Western countries, the Netherlands has to deal with an ageing society; the number of older adults (≥65 years) will rise from 3 million in 2016 to 4.7 million in 2060 (http://www.cbs.nl). Also, with an ageing population, the demand for healthcare increases, while health care budgets and manpower lag behind [1]. Consequently, even in countries that currently have a large publicly funded health care sector, such as the Netherlands, informal care is becoming more and more important [2]. However, the positive effect of reducing professional care must be balanced with the potential negative effects of informal caregiving [3]. In view of demographic changes such as reduced fertility, a growing instability of couple relationships, increasing labour market flexibility and higher rates of female employment, the question arises whether sufficient informal caregivers will be available in the future and how long the available informal caregivers will be able to maintain their expanding caregiving tasks [3–5].

To engage, educate and support informal caregivers adequately, more knowledge is needed with regard to informal caregiving. More specifically, two aspects of informal caregiving are often neglected in research. First, while a substantial number of studies have been conducted regarding informal caregiving among community-dwelling older adults, little is known about informal care in institutionalised long-term care (ILTC) [6–8]. Contrary to what one might expect informal caregiving often continues after admission to an ILTC facility [8, 9]; most informal caregivers make regular visits or even continue to perform care tasks such as personal care, managing money or buying groceries [8, 9]. In addition, informal caregivers often take on new tasks such as involvement in the residents’ council or communication with professional caregivers [8]. Second, irrespective of the setting, there is increasing evidence for the fact that informal caregiving may result in increased burden and subsequent negative health outcomes in caregivers [10]. However, informal caregivers may also experience positive aspects of care such as satisfaction, rewards, or enjoyment [11]. For example, in the study of de Boer et al. [12] only 7% of informal caregivers had no positive experiences in providing care. In addition, the study of Brouwer et al. [10] showed that half of the caregivers would become less happy, if somebody were to take over their caregiving tasks. Obviously, emphasizing the positive outcomes does not reduce the burden of informal caregiving directly, but a positive attitude regarding informal caregiving may positively influence the perception of care delivery [12–15], which may lead to improved health and wellbeing of both care receivers and caregivers and a more sustainable system of long-term care [9].

Consequently, the objectives of the current study were threefold: 1) to compare care receiver/caregiver characteristics and caregiver outcomes - at home and in ILTC; 2) to study the association between these characteristics and positive and negative caregiver outcomes (i.e., subjective burden and care-related quality of life) irrespective of the setting; and 3) to examine the moderating effect of the setting (at home vs. ILTC) on these associations.

## Methods

### Design and participants

For this cross-sectional study, we used The Older Persons and Informal Caregivers Survey Minimum DataSet (TOPICS-MDS; www.topics-mds.eu; [16]). TOPICS-MDS comprises a set of standardised questionnaires for collecting information on the physical and mental health and wellbeing of older adults and their primary informal caregivers. The data were collected in more than 60 different studies between 2010 and 2013 as part of The National Care for the Elderly Program, commissioned by the Dutch Ministry of Health, Welfare and Sport. A selection of 25 studies was used for the present analyses, as they collected TOPICS-MDS in both older adults and their informal caregivers. The study design features, sampling framework and survey mode varied between projects (See Additional file 1). For the identification of informal caregivers, a definition was provided by the TOPICS-MDS research group, but the method used to identify informal caregivers varied (See Additional file 1). The number of dyads ranged from 10 to 926 dyads. The TOPICS-MDS was assessed in writing or during an interview (See Additional file 1). We selected older adults who lived at home (*n* = 4277) and those who lived in ILTC facilities (*n* = 920). In total, cross-sectional data of 5197 older adults and their primary informal caregiver was available.

### Measurements

With regard to the care receiver characteristics, the following socio-demographic characteristics were taken into account: age, sex, ethnicity (i.e., native vs. non-native), educational level (i.e., low, middle, high), marital status (i.e., married/cohabiting vs. divorced/widowed/single), living situation (i.e., alone vs. with others). Furthermore, health-related characteristics were included in the analyses. First, multi-morbidity (17-item self-reported checklist) and disability (Katz-15; [17, 18]) of care receivers were included. Next, self-perceived health was assessed with the first question of the RAND-36 questionnaire [19] (i.e., *‘How is your health in general?’*). Finally, psychological wellbeing was measured with the 5-item subscale of the RAND-36 [19]. With regard to the caregiver characteristics, the following socio-demographic characteristics were included: age, sex, relationship with the care receiver (i.e., partner, child (in law), other) and whether the caregiver lived together with the care receiver (i.e., yes vs. no). Furthermore, health-related characteristics were included. Self-perceived health was measured using the same question as for the care receiver [19] (i.e., *‘How is your health in general?’*). Finally, caregiving-related characteristics were selected. More specifically, hours of informal caregiving were assessed by the question *‘How many hours of informal care they had delivered during the last week regarding (a) tasks in or around the house of the care receiver (e.g., cleaning, preparing meals), (b) personal care of the care receiver (e.g., dressing or eating) and (c) assistance with other activities (e.g., travelling outside the house, visiting friends or doctors or taking care of financial matters)’*. The number of hours was summed up with a theoretical maximum of 168 h/week [20]. Also, based on this question, the variation in caregiving tasks (<2 tasks vs. ≥2 tasks) was calculated. Finally, hours of support from other non-professional caregivers or volunteers were taken into account.

Finally, two caregiver outcomes were included. First, subjective burden was assessed with the self-rated burden scale [21]. Informal caregivers were asked to indicate how burdensome they experience caring for the care receiver. The answers range theoretically from 0 (not at all straining) to 100 (much too straining). Second, the Carer-QoL-7D [22] was used to measure seven dimensions of care-related quality of life (i.e. care-related fulfilment, relational problems with the care recipient, mental health problems, physical health problems, problems completing daily activities, financial security and social support). The dimensions can be used to derive a care-related quality of life summary score. Informal caregivers could describe their personal situation by responding ‘no’, ‘some’ or ‘a lot of’ for each dimension [23–25]. To generate a single summary score for the CarerQol-7D, the two positive items (care-related fulfilment and social support) were reversed and a set of weights [26] was applied to each level of the seven dimensions. The CarerQol-7D summary score represents a utility score for the care situation that ranges theoretically between ‘0’ (worst informal care situation) and 100 (best informal care situation).

More information about the TOPICS-MDS questionnaires can be found elsewhere (see Additional file 2).

### Statistical analysis

First, two groups of dyads were created based on the living situation of the care receiver: a) care receiver at home (whether or not living together with the caregiver); and b) care receiver living in an ILTC facility (nursing home/home for the elderly). Subsequently, the groups of dyads were described and compared with regard to a) care receiver/caregiver characteristics; and b) caregiver outcomes (study objective 1) using descriptive statistics and bivariate statistics (i.e. chi square tests and independent t-tests). With regard to the second study objective, the associations between care receiver/caregiver characteristics and caregiver outcomes were evaluated applying mixed model multi-level analyses, as data were clustered within individual studies. The two outcomes 1) subjective burden and 2) care-related quality of life were included as dependent variables in separate models.

Finally, to investigate the moderating effect of the setting (at home vs. ILTC) on the associations between care receiver/caregiver characteristics and caregiver outcomes (study objective 3) an interaction term (independent variable x setting) was included in the analyses. Living at home was coded as 1 and living in ILTC was coded as 2. Missing values were imputed by means of multiple imputations (except for categorical variables). To check for the robustness of the analyses, analyses were also conducted without imputation of missing values. For all statistical analyses the software package, SPSS for Windows, version 22.0, was used. An alpha level of .05 was considered as statistically significant.

## Results

### Characteristics of care receivers and caregivers and caregiver outcomes

Most characteristics of care receivers and caregivers and caregiver outcomes differed significantly between dyads at home and dyads in ILTC (see Table 1). Care receivers at home were younger and more often male than care receivers in ILTC facilities. Furthermore, they had better self-perceived health, fewer morbidities and lower levels of disability. However, psychological wellbeing was similar for the two groups of care receivers. Caregivers at home were mostly caring for either their parents or their partner. In contrast, ILTC caregivers were mostly caring for their parents and were consequently younger. The level of self-perceived health was significantly lower in caregivers at home compared to caregivers in ILTC. In addition, they delivered more caregiving hours than caregivers in ILTC, indicating a higher objective burden. Despite the higher level of objective burden at home, there was no significant difference between the groups with regard to caregivers’ subjective burden. However, caregivers’ care-related quality of life was significantly lower among caregivers caring for a care receiver at home compared to caregivers in ILTC.

**Table 1:** Care receiver/ caregiver characteristics and caregiver outcomes at home and in institutionalised long-term care (ILTC)

	Total (*n* = 5197)			At home (*n* = 4227)			ILTC (*n* = 920)			*p*-value
Care receiver characteristics										
Socio-Demographic Characteristics	n			n			n			
Age, mean (sd)	5197	80.7	(7.25)	4227	**79.6**	**(6.9)**	920	**85.7**	**(6.5)**	**<.001**
Female, %	5197	62		4277	**59**		920	**76**		**<.001**
Native, %	5190	94		4271	**93**		919	**96**		**.005**
Mean level of education	4615			3740			875			**<.001**
Low, %		44			**41**			**55**		
Middle, %		51			**52**			**43**		
High, %		5			**7**			**3**		
Marital status	5191			4273			918			**<.001**
Married/cohabiting, %		48			**54**			**20**		
Widowed/divorced/single, %		52			**46**			**80**		
Health-Related Characteristics	n			n			n			
Number of morbidities, mean (sd), *ranging from * **0** * to 17* ^a^	2,142^b^	4.0	(2.5)	1918 ^b^	**3.9**	**(2.5)**	224 ^b^	**4.6**	**(2.4)**	**<.001**
Disability, mean (sd), *ranging from * **0 ** *to 15* ^a^	4118 ^b^	5.9	(3.4)	3344 ^b^	**5.5**	**(3.2)**	774 ^b^	**7.8**	**(3.3)**	**<.001**
Self-perceived health, mean (sd), *ranging from 1 to * **5 ** ^a^	4334 ^b^	2.5	(.8)	3489 ^b^	**2.5**	**(.8)**	845 ^b^	**2.4**	**(.8)**	**<.001**
Psychological wellbeing, mean (sd), *ranging from 5 to * **30 ** ^a^	4096 ^b^	22.6	(4.3)	3303 ^b^	22.7	(4.2)	793 ^b^	22.4	(4.7)	.080
Caregiver characteristics										
Socio-Demographic Characteristics	n			n			n			
Age, mean (sd)	4563 ^b^	63.2	(12.5)	3680 ^b^	**64.0**	**(12.9)**	883 ^b^	**60.1**	**(10.4)**	**<.001**
Female, %	4081	69		3201	69		880	71		.188
Relationship with care receiver	5141			4235			906			**<.001**
Child (in law), %		51			**46**			**75**		
Partner, %		39			**46**			**10**		
Others, %		10			**8**			**15**		
Living together with care receiver, %	5154	51		4239	**50**		915	**6**		**<.001**
Health-Related Characteristics	n			n			n			
Self-perceived health, mean (sd), *ranging from 1 to * **5 ** ^a^	5050 ^b^	3.1	(1.0)	4148 ^b^	**3.1**	**(1.0)**	902 ^b^	**3.2**	**(1.0)**	**<.001**
Caregiving-Related Characteristics	n			n			n			
Objective burden, mean (sd), *ranging from 0 to 168 h*	3950 ^b^	17.4	(24.2)	3168 ^b^	**19.2**	**(25.5)**	782 ^b^	**9.0**	**(14.0)**	**<.001**
Tasks around the house (e.g., cleaning, preparing meals)		9.9	(16.3)		**11.2**	**(17.3)**		**4.0**	**(8.1)**	
Personal care (e.g., dressing or eating)		3.7	(9.4)		**3.8**	**(10.2)**		**1.5**	**(4.9)**	
Assistance with other activities (e.g., travelling, financial matters)		4.7	(8.5)		**4.9**	**(9.1)**		**3.5**	**(4.9)**	
Variation in caregiving tasks	3950 ^b^			3168 ^b^			782 ^b^			**<.001**
< 2 tasks, %		32			**31**			**39**		
≥ 2 tasks, %		68			**69**			**61**		
Hours support per week, mean (sd) *ranging from 0 to 168 h*	3950 ^b^	2.9	(7.8)	3168 ^b^	**3.0**	**(8.3)**	782 ^b^	**2.3**	**(5.2)**	**.032**
Caregiver outcomes	n			n			n			
Subjective burden, mean (sd), *ranging from * **0 ** *to 100* ^a^	4746 ^b^	41.9	(25.0)	3870 ^b^	42.2	(25.2)	876 ^b^	40.1	(24.2)	.134
Quality of life mean (sd), *ranging 0–* **100 ** ^a^	4074 ^b^	79.6	(15.0)	3287 ^b^	**78.9**	**(15.1)**	787 ^b^	**82.7**	**(14.0)**	**0.014**

Significant results (*p* < 0.05) are in bold

^a^ bold scores are most favourable scores

^b^ missing values imputed by multiple imputation

### Associations between care receiver/caregiver characteristics and caregivers’ subjective burden

Informal caregivers experienced significantly more subjective burden, if the care receiver was male (40.736 vs. 38.641) and married/cohabiting (40.283 vs. 38.733). In addition, caregivers’ subjective burden increased with more morbidities (ß = 1.090) and higher levels of disability (ß = 1.678) and decreased with better self-perceived health (ß = −2.990) and psychological well-being (ß = −.860) of the care receiver. With regard to the caregiver characteristics, being female (40.515 vs. 35.768), being the partner (40.534) and living together with the care receiver (40.323 vs. 38.722) was significantly associated with more subjective burden. In addition, the subjective burden decreased with better self-perceived health (ß = −5.868) of the caregiver and increased with more caregiving hours (ß = .224), more variation in caregiving tasks (42.811 vs. 31.994) and more support hours (ß = .157). Details are displayed in Table 2.

**Table 2:** Association between care receiver/ caregiver characteristics and caregivers’ subjective burden and care-related quality of life

	SUBJECTIVE BURDEN				QUALITY OF LIFE			
	*intercept*	*(se)*	*beta*	*(se)*	*intercept*	*(se)*	*beta*	*(se)*
Care receiver characteristics								
Socio-demographic characteristics								
Age	31.535	4.306	.098	.050	**66.173**	**3.473**	**.174**	**.033**
Sex								
Male	**40.736**	**1.578**	**−2.096**	**.713**	**77.394**	**1.718**	**4.542**	**.616**
Female	**38.641**	**1.536**	**2.096**	**.713**	**81.936**	**1.330**	**−4.542**	**.616**
Ethnicity								
Native	39.301	1.519	1.870	1.419	80.290	1.492	−1.263	.897
Non-native	41.171	2.021	−1.870	1.419	79.026	1.466	1.264	.897
Education								
Low vs. middle	39.158	1.541	.038	.768	80.540	1.587	−.384	.438
Middle vs. high	39.197	1.525	1.067	1.632	80.156	1.579	−.556	.934
High vs. low	40.263	2.104	−1.105	1.653	79.600	1.729	.940	.944
Marital status								
Married/cohabiting	**40.283**	**1.574**	**−1.551**	**.705**	**78.089**	**1.637**	**3.962**	**.538**
Widowed/divorced/single	**38.733**	**1.559**	**1.551**	**.705**	**82.051**	**1.362**	**−3.962**	**.538**
Health-related characteristics								
Number of morbidities, *ranging from * **0 ** *-17* ^a^	**35.132**	**1.686**	**1.090**	**.206**	**82.555**	**1.691**	**−.588**	**.140**
Disability, *ranging from * **0 ** *to 15* ^a^	**30.297**	**1.435**	**1.678**	**.106**	**81.543**	**1.852**	**−.240**	**.097**
Self-perceived health, *ranging from 1 to * **5 ** ^a^	**46.662**	**1.869**	**−2.990**	**.427**	**76.486**	**1.447**	**1.549**	**.270**
Psychological wellbeing, *ranging from 5 to * **30 ** ^a^	**58.879**	**2.366**	**−.860**	**.081**	**71.403**	**1.628**	**.391**	**.051**
Caregiver characteristics								
Socio-demographic characteristics								
Age	40.519	2.596	−.014	.031	**88.353**	**1.435**	**−.121**	**.025**
Sex								
Male	**35.768**	**1.804**	**4.747**	**.829**	**83.035**	**1.164**	**−3.053**	**.507**
Female	**40.515**	**1.732**	**−4.747**	**.829**	**79.982**	**1.102**	**3.053**	**.507**
Relationship with care receiver								
Child vs. partner	39.409	1.557	1.124	.767	**82.036**	**1.284**	**−5.210**	**.712**
Partner vs. others	**40.534**	**1.579**	**−4.856**	**1.257**	**76.825**	**1.741**	**6.165**	**.752**
Others vs. child	**35.678**	**1.848**	**3.731**	**1.251**	82.991	1.649	−.955	.772
Living situation								
Living together	**40.323**	**1.591**	**−1.602**	**.737**	**77.360**	**4.676**	**4.977**	**.606**
Not living together	**38.722**	**1.568**	**1.602**	**.737**	**82.337**	**1.330**	**−4.977**	**.606**
Health-Related Characteristics								
Self-perceived health, *ranging from 1 to * **5 ** ^a^	**57.466**	**1.863**	**−5.868**	**.354**	**61.606**	**1.458**	**6.063**	**.229**
Caregiving-Related Characteristics								
Objective burden, *ranging from 0 to 168*	**35.821**	**1.435**	**.224**	**.015**	**81.925**	**1.235**	**−.105**	**.019**
Variation in caregiving tasks								
< 2 tasks	**31.994**	**1.515**	**10.818**	**.731**	**82.329**	**1.147**	**−3.061**	**.797**
≥ 2 tasks	**42.811**	**1.441**	**−10.818**	**.731**	**79.268**	**1.638**	**3.061**	**.797**
Support (hours), *ranging from 0 to 168*	**39.005**	**1.502**	**.157**	**.046**	80.144	1.410	.031	.040

Significant results based on mixed-model multilevel analyses (*p* < 0.05) are in bold

^a^ bold scores are most favourable scores

Informal caregivers experienced a significantly higher care-related quality of life, if the care receiver was older (ß = .174), female (81.936 vs. 77.394) and widowed/divorced/single (82.051 vs. 78.089). In addition, caregivers’ quality of life decreased with more morbidities (ß = −.588) and higher levels of disability (ß = −.240) and increased with better self-perceived health (ß = 1.549) and psychological well-being (ß = .391) of the care receiver. With regard to the caregiver characteristics, being younger (ß = −.121) and being male (83.035 vs. 79.982) was significantly associated with higher care-related quality of life. In contrast, being the partners and living together (77.360 vs. 82.337) with the care receiver decreased the caregivers’ quality of life. In addition, care-related quality of life increased with better self-perceived health (ß = 6.063) and decreased with more caregiving hours (ß = −.105) and more variation in caregiving tasks (79.268 vs. 82.329). Details are displayed in Table 2.

### Moderating effect of the setting (at home vs. ILTC) on these associations

The results, with regard to the moderating effect of the setting (at home vs. ILTC) on the associations between care receiver/caregiver characteristics and caregiver outcomes, are presented in Table 3. While the increasing age of the care receiver at home is associated with an increasing subjective burden (ß = .146), it was the other way around in ILTC (ß = −.192). Furthermore, when comparing both settings, the caregivers’ subjective burden at home increased sharply with the increasing disability of the care receiver (ß = 1.946 vs. ß = 1.068) and there was more variation in caregiving tasks than in ILTC (at home: 31.142 vs. 42.861; ILTC: 35.763 vs. 42.939). Also, the positive association between caregivers’ self-perceived health and care-related quality of life is stronger in caregivers caring for a care receiver at home compared to caregivers in ILTC (ß = 6.305 vs. ß = 4.939).

**Table 3:** Significant interaction effects between with regard to caregivers’ subjective burden and care-related quality of life

	SUBJECTIVE BURDEN				QUALITY OF LIFE			
	*intercept*	*(se)*	*beta*	*(se)*	*intercept*	*(se)*	*beta*	*(se)*
Care receiver characteristics								
Socio-demographic characteristics								
Age*, institutionalised long-term care*	56.633	(11.125)	−.192	(.130)				
Age*, at home*	27.567	(4.763)	.146	(.056)				
Health-Related Characteristics								
Disability *(ranging from * **0 ** *to 15* ^a^ *) institutionalised long-term care*	32.034	(2.596)	1.068	(.245)				
Disability (*ranging from * **0 ** *to 15* ^a^ *), at home*	29.517	(1.433)	1.946	(.121)				
Caregiver characteristics								
Health-Related Characteristics								
Self-perceived health (*ranging from 1 to * **5 ** ^a^)*, institutionalised long-term care*					66.226	(2.207)	4.939	(.462)
Self-perceived health (*ranging from 1 to * **5 ** ^a^)*, at home*					60.653	(1.433)	6.305	(.267)
Caregiving-Related Characteristics								
< 2 tasks*, institutionalised long-term care*	35.763	(2.029)	7.175	(1.654)				
≥ 2 tasks*, institutionalised long-term care*	42.939	(1.875)	−7.175	(1.654)				
< 2 tasks*, at home*	31.142	(1.546)	11.719	(.813)				
≥ 2 tasks*, at home*	42.861	(1.455)	−11.719	(.813)				

Only significant interaction terms (*p* < 0.05) are displayed

^a^ bold scores are most favourable scores

## Discussion

With regard to the *first* study objective, our study showed that care receivers at home were younger and in better health than care receivers in ILTC. However, both groups of care receivers reported a similar level of psychological wellbeing. Consequently, admission to an ILTC facility does not necessarily reduces the well-being of care receivers. This is line with the study of Beerens et al. [27] that showed that care receivers’ quality of life is comparable at home and in ILTC. Furthermore, our study showed that caregivers in ILTC experienced lower objective burden compared to caregivers at home. However, caregivers in ILTC still delivered a considerable number of caregiving hours (on average 9 h/week), which contributes to the evidence that informal caregiving often continues after admission to an ILTC [8, 9]. This is in line with previous studies [6, 8, 9] that have shown that caregivers in ILTC still experience high levels of subjective burden. Consequently, it is understandable that ILTC caregivers in our study experienced a comparable subjective burden to caregivers at home, even though they devote significantly less time to their caregiving tasks. Our study also indicated that caregivers’ care-related quality of life was somewhat lower at home compared to ILTC, which may be due to the fact that caregivers at home were mostly caring for either their parents or their partner. In contrast, in ILTC most caregivers were caring for their parents and were consequently younger and in better health. However, it is a remarkable finding that caregivers in both settings experienced relatively high levels of care-related quality of life despite their burden [3, 28]. Obviously, positive experiences may act as a buffer against the stress-related consequences of informal caregiving [4]. Nevertheless, it is important to engage, educate and support informal caregivers, as they are more likely to experience poor health and decreased wellbeing than non-caregivers [29–31]. This is especially true when their caregiving tasks exceed their psychological and social resources to cope with the care needs of the care receiver [4].

With regard to the *second* study objective, several significant associations were found between care receiver/caregiver characteristics and caregiver outcomes. Higher subjective burden and lower care-related quality of life in caregivers were associated with the following care receiver characteristics: being male, married/cohabiting, and poor health-related characteristics. In addition, caregivers experienced an increased subjective burden and a decrease in care-related quality of life, if they were female, lived together with the care receiver, had higher objective burden and lower self-perceived health. These risk factors are in line with previous research [32]. In addition, support from other informal caregivers or volunteers was associated with higher subjective burden. Obviously, support is an indicator for a burdensome caregiving situation. Increasing professionals’ awareness regarding informal caregivers’ risks and providing them with tools and information to adequately support and involve them in care delivery is highly important to adequately engage, educate and support informal caregivers [4] so that they can persevere in their caregiving task for longer [3].

With regard to the *third* study objective, our study showed that whereas the subjective burden increased with the care receivers’ age at home, it decreased with age in ILTC. This is maybe due to the fact that informal caregivers in ILTC experience more support from professional caregivers, who take over the more challenging, intensive, and essential caregiving tasks, while informal caregivers provide voluntary, less intensive and less onerous help [33]. Furthermore, analyses have shown that higher levels of disability of the care receiver and more variation in caregiving tasks had more impact at home on subjective burden than in ILTC. Also the association between lower levels of caregivers’ self-perceived health and lower care-related quality of life is stronger at home than in ILTC. This is maybe due to the fact that informal caregivers at home might not feel capable anymore to care for the care receiver [34], while informal caregivers in ILTC receive support from professional caregivers [33].

### Strengths and limitations

Although many informal caregiving studies exist the strength of this study is the large data set of dyads. Furthermore, data from care receivers and caregivers in two long-term care settings (i.e., at home and in ILTC) are collected using the same outcome measures. However, some limitations of our study must be acknowledged as well. First, the care receivers and caregivers in the TOPICS-MDS database showed some variation regarding sampling frame, inclusion criteria, study design, sample size, and data collection method. This variation may influence the generalisability of the results. Nonetheless, these pooled data probably reflect reality better than data from a single project. Secondly, although a definition was provided by the TOPICS-MDS research group for the identification of informal caregivers, only 16 out of 25 studies made use of this definition. Consequently, it is not clear whether the informal caregivers of the other studies are fully comparable with the sample of these 16 studies. Thirdly, a further limitation of this study is the relatively high nonresponse on some items (see Table 1). Nevertheless, robustness checks without imputed missing values did not alter the conclusions (results not displayed). Fourthly, the TOPICS-MDS database was not specifically designed for the specific aims of this study. Consequently, operationalization of some variables was pragmatic and some relevant variables might have been missed. For example, data about other caregiver responsibilities (e.g., household chores, employment) or adaptive resources (e.g., coping behaviour, finances) were not assessed in the TOPICS-MDS. Finally, this study is based on cross-sectional data and the results have to be interpreted with caution, as no causal relationship can be proven.

In conclusion, this study provides insight into the objective and subjective burden and care-related quality of life of informal caregivers - at home and in ILTC. While a substantial number of studies have been conducted among community-dwelling older adults and their informal caregivers, little was known about informal care in ILTC, especially with regard to caregiving hours provided (objective burden). The direct comparison between the two settings is also novel. Furthermore, this study showed that despite significantly less caregiving hours in ILTC, caregivers in ILTC experienced a comparable level of subjective burden to caregivers at home. This finding implies that a reduction in objective burden resulting from an admission to an ILTC facility does not necessarily have to lead towards a relief of subjective caregiving burden. However, this study has also shown that informal caregivers at home are still most at risk of experiencing higher subjective burden and lower care-related quality of life than caregivers in ILTC.

### Implications

This study results in several implications for policy and practice:

1. Policy makers and professional caregivers have to take into account that caregiving does not stop with admission to an ILTC facility. Consequently, both settings need an informal caregiving policy that facilitates complementary service delivery, which is tailored to the individual needs and preferences of care receivers and informal caregivers. Consequently, it is essential that professional caregivers assess these needs and evaluate the provided services on a regular base. It is likely that care receiver and caregiver needs are different at home care and in ILTC due to their individual characteristics. For example, this study has shown that caregivers at home often care for their partner. In contrast, in ILTC most caregivers were caring for their parents. It is obvious that children have other needs than partners due to differences in age, health status and additional responsibilities (e.g., employment). Furthermore, admission to an ILTC facility also leads towards a shift in caregiver tasks and responsibilities. However, it is not clear whether these tasks are expected by professional caregivers or whether informal caregivers by themselves feel obligated to participate in these tasks. It is important that professional and informal caregivers exchange their expectations regarding these tasks and responsibilities to avoid an increase in subjective burden and a decrease in care-related quality of life.
2. In this study, several significant associations were found between care receiver/caregiver characteristics and caregiver outcomes. Higher subjective burden and lower care-related quality of life in caregivers were associated with the following care receiver characteristics: being male, married/cohabiting, and poor health-related characteristics. In addition, caregivers experienced an increased subjective burden and a decrease in care-related quality of life, if they were female, lived together with the care receiver, had higher objective burden and lower self-perceived health and received support from other informal caregivers or volunteers was associated with higher subjective burden. Some of these associations were stronger for dyads at home compared to dyads in ILTC. Professional caregivers should pay special attention to these groups of informal caregivers, for example by assessing these (and eventual other) risk factors to eventually offer proactive interventions (e.g., psychological or social support) to reduce or even prevent these outcomes. Furthermore, at home professional caregivers may have an important role in monitoring and discussing the need for an ILTC admission.
3. Despite their objective and subjective burden, caregivers in both settings experienced relatively high levels of care-related quality of life. Obviously, positive experiences may act as a buffer against the stress-related consequences of informal caregiving. A more balanced perspective on informal caregiving is suggested, which aims to reduce the negative outcomes and emphasise the positive outcomes at the same time. Consequently, professional caregiver should stimulate informal caregivers to reflect on their positive experiences with being a caregiver. In addition, it is important to acknowledge their efforts by giving them compliments for their executed caregiving tasks.

## Conclusion

This study contributes to the evidence that Informal caregiving does not stop with admission to an ILTC facility. Caregivers in both settings experienced high burden, and caregivers in ILTC still delivered a considerable number of caregiving hours. Several care receiver/caregiver characteristics were identified who were associated with an increase in burden and/or a decrease in care-related quality of life. Some of these associations were stronger for dyads at home compared to dyads in ILTC indicating a higher risk for this subgroup. To support caregivers in their caregiving tasks both settings need an informal caregiving policy, which is (1) tailored to the individual characteristics of care receivers and caregivers; (2) pays attention to the identified risk groups; and (3) reduces the negative caregiver outcomes and emphasizes the positive outcomes at the same time.

## Additional files

Additional file 1: Background information included studies. ^a^ The TOPICS-MDS research group provided the following definition of informal caregivers: those who deliver, voluntarily and unpaid on a structural basis, care for people with physical, mental or psychological limitations in their family, household or social network because the care-receivers’ health prohibits them from doing certain things themselves. (DOCX 50 kb)

Additional file 2: Background information about TOPICS-MDS questionnaire. ^a^ Only items that were relevant for this study are displayed. (DOCX 45 kb)

## Abbreviations

ILTC: institutionalised long-term care
SPSS: Statistical package for the social sciences
TOPICS-MDS: The Older Persons and Informal Caregivers Survey Minimum DataSet
