# Supplementary material for: Positive and negative outcomes of informal caregiving at home and in institutionalised long-term care: a cross-sectional study
Source: BMC Geriatr. 2017 Oct 10;17:232. doi: 10.1186/s12877-017-0620-3 (PMC5635563; doi:10.1186/s12877-017-0620-3)
Supplement: Supplementary file 1 — Background information included studies. a The TOPICS-MDS research group provided the following definition of informal caregivers: those who deliver, voluntarily and unpaid on a structural basis, care for people with physical, mental or psychological limitations in their family, household or social network because the care-receivers’ health prohibits them from doing certain things themselves. (DOCX 50 kb) [file 12877_2017_620_MOESM1_ESM.docx]

| **Additional file 1. Background information included studies** | | | | | | | | | |
| --- | --- | --- | --- | --- | --- | --- | --- | --- | --- |
| **No.** | **Design** | **N dyads** | **Sampling frame** | **Main inclusion criterion care receiver** | **Method of data collection** | **Informal caregiver definition^a^ used** | **Informal caregiver identified, by whom?** | **Informal caregiver identified, how?** | **References** |
| 1 | Prospective design | 500 | nursing home/ general population | age ≥65 yrs | unknown | unknown | unknown | unknown | unknown |
| 2 | RCT | 300 | hospital setting | frailty (ISAR-HP); age ≥65 yrs | written | yes | care receiver | face-to-face | Buurman et al. BMC Health Serv Res, 2010 |
| 3 | Quasi-experimental | 926 | hospital setting | frailty (ISAR-HP, NPI-Q and MMSE); age ≥65 yrs | interview | yes | care receiver | face-to-face | Asmus-Szepesi et al. BMC Geriatr, 2011 |
| 4 | Prospective design | 289 | primary care | frailty (Easycare-TOS); age ≥70 | interview | yes | general practitioner/care receiver | registration/ face-to-face | unknown |
| 5 | Validation study | 102 | primary care | age ≥70 | interview | yes | general practitioner / care receiver | registration/ face-to-face | Van Kempen et al, J Clin Epidemiol, 2014 |
| 6 | RCT | 57 | primary care | frailty (ISCOPE questionnaire); age ≥75 yrs | written | yes | care receiver | face-to-face | Blom et al, Age Ageing, 2016 |
| 7 | RCT | 91 | primary care | age ≥75 yrs; frailty (instrument unknown) | written | yes | care receiver | face-to-face | unknown |
| 8 | Prospective design | 377 | residential care facility | all residents | written | yes | other (home for the elderly) | registration | Poot et al, Plos ONE 2016 |
| 9 | RCT | 222 | primary care | frailty (GFI); age ≥70 yrs | written | yes | care receiver | by telephone | Metzelthin et al, BMC Health Serv Res, 2010 |
| 10 | Quasi-experimental | 199 | primary care | frailty (GFI); age ≥75 yrs | interview/ written | yes | care receiver | face-to-face | Fabbricotti et al, BMC Geriatr, 2013 |
| 11 | Pilot study | 10 | hospital setting | Frailty (instrument unknown); age ≥70 yrs | written | no | care receiver | face-to-face | Bakker et al, J Am Soc 2013 |
| 12 | RCT | 144 | residential care facility | other (stroke); age ≥65 yrs | written | yes | care receiver | face-to-face | Vluggen et al, BMC Neurol 2012 |
| 13 | Quasi-experimental design | 496 | general population | other (dementia) | interview/ written | yes | care receiver/ general practitioner / other | by telephone, written, registration | Vroomen et al, BMC Health Serv Res 2012 |
| 14 | Quasi-experimental design | 112 | general population | age ≥65 yrs | written | no | care receiver | face-to-face | unknown |
| 15 | Prospective design | 522 | primary care | other (dementia) | unknown | no | other (case manager) | written | Peeters et al, BMC Geriatr 2016 |
| 16 | Quasi-experimental | 50 | primary care | other (medical unexplained symptoms); age ≥65 yrs | written | yes | care receiver | face-to-face | Hanssen et al, Fam Pract 2016 |
| 17 | Quasi-experimental | 64 | general population | frailty (TFI); age ≥70 yrs | interview/ written | yes | care receiver | face-to-face | Cramm et al, BMC Res Notes, 2011 |
| 18 | Quasi-experimental? | 111 | residential care facility | other (dementia) | interview | yes | other (day care centre) | by telephone | unknown |
| 19 | Cross-sectional design | 12 | general population | age ≥70 yrs | written | no | care receiver | face-to-face | Pijpers et al, J Housing Elderly 2016. |
| 20 | RCT | 194 | primary care | frailty (Easycare-TOS); age ≥70 yrs | interview | yes | general practitioner / care receiver | registration/ face to face | Ruikes et al, BMC Family Practice 2012 |
| 21 | Quasi-experimental | 41 | general population | age ≥75 yrs | written | no | care receiver | written | Janssen et al, Maastricht University, 2015 (rapport). |
| 22 | Cross-sectional design | 55 | residential care facility | all residents | written | no | other (nursing home) | registration | Daamen et al, BMC Getriatrics, 2015 |
| 23 | Quasi-experimental | 158 | hospital setting | frailty (ISAR-HP, NPI-Q, MMSE); age ≥65 yrs | written | yes | care receiver | face-to-face | Asmus-Szepesi et al. BMC Geriatr 2011 |
| 24 | Pre-posttest | 89 | hospital setting | other (expected stay >48 hrs; age ≥ 70 yrs | written | no | care receiver | face-to-face | Bakker et al, American Journal of Surgery 2014 |
| 25 | Pre-posttest | 76 | hospital setting | other (expected stay >48 hrs; age ≥ 70 yrs | written | no | care receiver | face-to-face | Bakker et al, American Journal of Surgery 2014 |
| RCT=randomised controlled trial; ISAR-HP= Identification of Seniors at Risk-Hospitalized Patient; NPI-Q=Neuropsychatric Inventory Questionnaire; MMSE=Mini-Mental State Examination  Easycare-TOS=Easy-Care-Two-step Older persons Screening; ISCOPE=Integrated Systematic Care for Older People; GFI=Groningen Frailty Indicator; TFI=Tilburg Frailty Indicator.  ^a^ The TOPICS-MDS research group provided the following definition of informal caregivers: those who deliver, voluntarily and unpaid on a structural basis, care for people with physical, mental or psychological limitations in their family, household or social network because the care-receivers’ health prohibits them from doing certain things themselves. | | | | | | | | | |

c
