# Supplementary material for: Positive and negative outcomes of informal caregiving at home and in institutionalised long-term care: a cross-sectional study
Source: BMC Geriatr. 2017 Oct 10;17:232. doi: 10.1186/s12877-017-0620-3 (PMC5635563; doi:10.1186/s12877-017-0620-3)
Supplement: Supplementary file 2 — Background information about TOPICS-MDS questionnaire. a Only items that were relevant for this study are displayed. (DOCX 45 kb) [file 12877_2017_620_MOESM2_ESM.docx]

| **Additional file 2. Background information about TOPICS-MDS questionnaire^a^** | | |
| --- | --- | --- |
| **Variable** | **Operationalisation** | **Psychometric properties** |
| **Care receiver characteristics** | | |
| Age | Date of birth | N/A |
| Female | Gender | N/A |
| Ethnicity | In which country were your born? | N/A |
| Education | What is the highest level of education that you have completed?   - Fewer than 6 years of primary school - 6 years of primary school, lom school, mlk school (special education) - More than primary school / primary school without further completed - education - Vocational school - Mulo / mms / mavo / secondary professional education - Hbs / gymnasium / atheneum (university entrance level) - University / tertiary education | N/A |
| Marital status | What is your marital status?   - Married - Divorced - Widow / widower / partner deceased - Unmarried - Long-term cohabitation, unmarried | N/A |
| Number of morbidities | Place a tick next to the illnesses and conditions that you have at the moment or have had in the past 12 months. You can select more than one answer.   - Diabetes - Stroke, cerebral haemorrhage (bleed in the brain), cerebral infarction (blocked blood vessel in the brain) or TIA - Heart failure - A type of cancer (malignant condition) - Asthma, chronic bronchitis, lung emphysema or COPD - Involuntary loss of urine (incontinence) - Wearing of the joints (arthrosis, degenerative arthritis) of hips or knees - Loss of bone tissue (osteoporosis) - Broken hip - Broken bones other than a broken hip - Dizziness with falling - Prostate symptoms caused by benign prostate enlargement - Depression - Anxiety / panic disorder - Dementia - Hearing problems - Problems with vision | N/A |
| Disability | To measure disability a modified version of the Katz Index of Independence Basic Activities of Daily Living (ADL) and Instrumental Activities of Daily Living (IADL) was used([Katz, Ford, Moskowitz, Jackson, & Jaffe, 1963](#_ENREF_3); [Lawton & Brody, 1969](#_ENREF_4)). Respondents were asked if assistance is required for six basic functions (i.e. bathing; dressing; eating; toileting; use of incontinence products; getting up from a chair) and seven instrumental functions (i.e. grooming; use of telephone; travelling; grocery shopping; meal preparation, household tasks; taking medications; financial management). In addition, to measure mobility respondents were asked if assistance was required while walking ([Weinberger et al., 1992](#_ENREF_11)). | Metrics to assess ADL and IADL, such as the Katz Index, have been administered in a variety of geriatric populations ([Palmer & Harley, 2012](#_ENREF_7)) and has been shown to produce reliable results irrespective of completion by a respondent or a proxy ([Buurman, van Munster, Korevaar, de Haan, & de Rooij, 2011](#_ENREF_2); [Weinberger et al., 1992](#_ENREF_11)). |
| Self-perceived health | Item RAND-36: How is your health in general? ([Van der Zee & Sanderman, 2002](#_ENREF_9))   - Excellent - Very good - Good - Reasonable - Poor | N/A |
| Psychological wellbeing | Psychological wellbeing was measured using the RAND-36 mental state subscale ([Van der Zee & Sanderman, 2002](#_ENREF_9)). The sub-scale is comprised of five questions asking respondents how often in the past four weeks they have felt (1) very nervous, (2) calm and peaceful, (3) down-hearted and blue, (4) happy and (5) so down in the dumps nothing could cheer [them] up. | The Rand-36 mental state sub-scale reliably measures a unidimensional concept of mental state ([Moorer, Suurmeije, Foets, & Molenaar, 2001](#_ENREF_6)). |
| **Caregiver characteristics** | | |
| Age | Date of birth | N/A |
| Female | Gender | N/A |
| Relationship with care receiver | What is the relationship with your loved one?  I am his/ her   - Husband/ wife/ life partner - Sister(-in-law), brother (-in-law) - Daughter (-in-law)/ son-(-in-law) - Other, namely: | N/A |
| Living together with care receiver | Do you live with your loved one?   - Yes - No | N/A |
| Self-perceived health | Item RAND-36: How is your health in general?   - Excellent - Very good - Good - Reasonable - Poor | N/A |
| Objective burden | How many hours of informal care they had delivered during the last week regarding   1. tasks in or around the house of the care receiver (e.g., cleaning, preparing meals) 2. personal care of the care receiver (e.g., dressing or eating) 3. assistance with other activities (e.g., travelling outside the house, visiting friends or doctors or taking care of financial matters)’ | Despite the potential for recall bias, retrospective reports of hours of informal care can yield valid and reliable results in cross-sectional studies if adjustments for multi-tasking are included in the analysis ([Van den Berg & Spauwen, 2006](#_ENREF_8)). |
| Hours support per week | Does your loved one receive help from other caregivers or volunteers besides you?   - No - Yes, namely _ _ _ hours per week | N/A |
| **Caregiver outcomes** | | |
| Subjective burden | Subjective burden was assessed with the self-rated burden scale ([Van Exel et al., 2004](#_ENREF_10)). Informal caregivers were asked to indicate how burdensome they experience caring for the care receiver. | The self-rated burden scale is more feasible and at least as valid than the longer and more complex instruments for the assessment of caregiver burden ([Van Exel et al., 2004](#_ENREF_10)). |
| Quality of life | The Carer-QoL-7D ([Brouwer, van Exel, van Gorp, & Redekop, 2006](#_ENREF_1)) was used to measure care-related quality of life. It includes seven attributes: care-related fulfilment; relational problems with the care recipient; mental health; time management; financial security; social support; and physical health. | CarerQol-7D has proven validity and applicability in a variety of settings. Due to minor differential reporting, pooling data collected using mixed administration modes should be interpreted with caution ([Lutomski et al., 2015](#_ENREF_5)). |
| *^a^ Only items that were relevant for this study are displayed* | | |

**References**

Brouwer, W. B., van Exel, N. J., van Gorp, B., & Redekop, W. K. (2006). The CarerQol instrument: a new instrument to measure care-related quality of life of informal caregivers for use in economic evaluations. *Qual Life Res, 15*(6), 1005-1021.

Buurman, B. M., van Munster, B. C., Korevaar, J. C., de Haan, R. J., & de Rooij, S. E. (2011). Variability in measuring (instrumental) activities of daily living functioning and functional decline in hospitalized older medical patients: a systematic review. *J Clin Epidemiol, 64*(6), 619-627.

Katz, S., Ford, A. B., Moskowitz, R. W., Jackson, B. A., & Jaffe, M. W. (1963). Studies of illness in the aged: The index of ADL: A standardized measure of biological and psychosocial function. *JAMA, 185*(12), 914-919.

Lawton, M., P., , & Brody, E. M. (1969). Assessment of older people: self-maintaining and instrumental activities of daily living. *Gerontologist, 9*, 179-186.

Lutomski, J. E., van Exel, N. J. A., Kempen, G. I. J. M., Moll van Charante, E. P., den Elzen, W. P. J., Jansen, A. P. D., . . . Melis, R. J. F. (2015). Validation of the Care-Related Quality of Life Instrument in different study settings: findings from The Older Persons and Informal Caregivers Minimum DataSet (TOPICS-MDS). *Qual Life Res, 24*, 1281-1293.

Moorer, P., Suurmeije, T. P., Foets, M., & Molenaar, I. W. (2001). Psychometric properties of the RAND-36 among three chronic diseases (multiple sclerosis, rheumatic diseases and COPD) in The Netherlands. *Qual Life Res, 10*(7), 637-645.

Palmer, M., & Harley, D. (2012). Models and measurement in disability: an international review. *Health Policy Plan, 27*(5), 357-364.

Van den Berg, B., & Spauwen, P. (2006). Measurement of informal care: an empirical study into the valid measurement of time spent on informal caregiving. *Health Econ, 15*(5), 447-460.

Van der Zee, K., & Sanderman, R. (2002). Het meten van de algemene gezondheidstoestand met de Rand-36: een handleiding

Van Exel, N. J., Scholte op Reimer, W. J., Brouwer, W. B., van den Berg, B., Koopmanschap, M. A., & van den Bos, G. A. (2004). Instruments for assessing the burden of informal caregiving for stroke patients in clinical practice: a comparison of CSI, CRA, SCQ and self-rated burden. *Clin Rehabil, 18*(2), 203-214.

Weinberger, M., Samsa, G. P., Schmader, K., Greenberg, S. M., Carr, D. B., & Wildman, D. S. (1992). Comparing proxy and patients' perceptions of patients' functional status: results from an outpatient geriatric clinic. *J Am Geriatr Soc, 40*(6), 585-588.
